# The Youth Patient and Public Involvement Café—A youth‐led model for meaningful involvement with children and young people

**DOI:** 10.1111/hex.13597

**Published:** 2022-09-05

**Authors:** Abigail Thomson, Edward Peasgood, Sam Robertson

**Affiliations:** ^1^ Research and Development Department, Sussex Education Centre Sussex Partnership NHS Foundation Trust Brighton UK; ^2^ Department of Experimental Psychology The University of Oxford Oxford UK; ^3^ East Sussex Community Voice CIC Eastbourne UK; ^4^ East Sussex County Council (Children's Services) Eastbourne UK

**Keywords:** children and young people, mental health, patient and public involvement, PPI, research

## Abstract

**Introduction:**

There are few meaningful frameworks or toolkits that exist for involvement with young people. Coproduction is a more recent patient and public involvement (PPI) approach that emphasizes the importance of power‐sharing, to set young people as equal partners in the research process. This paper explores the successes and challenges encountered by one coproduced PPI space for young people.

**Methods:**

This paper is written by a team of young people who developed and worked on the Youth PPI Café over a period of 18 months. It explores how we developed a youth‐led space for involvement in research. The authors have reflected on their experiences, providing examples of how youth PPI and coproduction were delivered in the NHS, in practice.

**Results:**

By working ‘with’ young people, rather than ‘for’ them, we offer insights into the successes and challenges of an entirely youth‐led involvement space. Despite being effective in shaping mental health research for children and young people, we faced challenges with tokenism, resourcing and diversity and inclusion.

**Conclusions:**

Involving youth meaningfully in research has the potential to inform studies at a macro‐ and microlevel, enabling positive change within research and within the systems that support young people.

**Patient or Public Contribution:**

Young people aged 16–24 years with lived experience were included at every stage of this project, from formulation to the delivery and development of the group, to the preparation of this manuscript and its dissemination. Sussex Partnership NHS Foundation Trust's charity ‘Heads On’ provided funding for this study.

## 1 INTRODUCTION

Mental health research carried out ‘with’ or ‘by’ members of the public, rather than ‘to’, ‘for’ or ‘about’ them is now widely encouraged in the United Kingdom.^1^ This process, most commonly referred to as patient and public involvement (PPI), includes service users and carers, or ‘members of the public’, as active collaborators in the research process. PPI has a range of benefits and, when carried out meaningfully, has the potential to transform mental health services and interventions.^2^ Yet, despite a growing acknowledgement of the importance of PPI within the field of mental health research, there remains a need to address the lack of diversity that exists within research and involvement.^3^, ^4^

Young people in particular have been identified as an under‐served group, both within services and research.^4^ Traditionally, children and young people (CYP) were viewed as ‘hard to reach’, lacking in what many believed to be a necessary understanding of the research processes.^5^ Though this view is changing, and there are more opportunities for young people to become involved in research today,^6^ there are few meaningful frameworks that exist for involvement with CYP. As a result, PPI with CYP is often delivered and embedded in practice in a way that is tokenistic or lacking in effectiveness.^7^ In addition, there have been few efforts to increase the accessibility of PPI for CYP from minority or low socioeconomic backgrounds, who, as such, remain heavily marginalized and under‐served in research.^8^

### 1.1 Meaningful and effective involvement in research—The Youth PPI Café

Increasingly more research is being carried out within the field of child and adolescent mental health to combat the growing prevalence of mental health problems within this population.^9^ To be effective and meaningful, such research should seek to involve a diverse collective of CYP from formulation to dissemination—in generating, and defining the research questions, in developing the methodology, collecting and analysing the data and in the dissemination of key findings amongst their peers. Such a process should set young people as equal partners in the research process and engage them in what should be a mutualistic process of involvement.^10^ Sussex Partnership NHS Foundation Trust's (SPFT) ‘Youth PPI Café’ sought to facilitate the establishment of this space, through the creation of a coproduced, collaborative peer‐based involvement network.

The initial aim of this space was to explore some of the common challenges that CYP face that might impact their involvement in research. There was a recognition that a young person's experience of mental health problems and their subsequent treatment are different from that of an adult. For example, the unique pressures that they face, including restrictions on capacity to give consent, legal frameworks that can disempower, the need for parental involvement, paternalistic attitudes by adults to ‘protect’ but that in reality prevent CYP from being able to give voice to their concerns, all can have a significant impact on their experience as a service user, and their ability to engage with research. The Youth PPI Café sought to understand the experience of being a young person, to best connect with SPFT's young service users and engage them in research. Following this, and through working collaboratively with CYP, we set out to create a safe, peer‐led space where CYP with lived experience could inform and develop current research centred around child and adolescent mental health. The Youth PPI Café set out to offer CYP the opportunity to use their unique lived experience to guide clinicians and researchers to take research and service development projects forward and implement them in practice. We aimed to co‐develop a programme of meaningful research ‘with’ CYP, rather than ‘for’ them.

### 1.2 For young people, by young people

Grounded in service user‐led practices, the Youth PPI Café was created by young people, for young people. With the key aim of establishing a model of meaningful involvement with CYP in research and initiating an improvement in youth involvement practices in research, it was important that the space created allowed us (as facilitators) to support and empower CYP to consult on studies, to take on an active role in the development of the Youth PPI Café and to become champions to implement and grow research. Philosophically, this has much in common with the coproduction of knowledge^11^ and has the potential to empower young people and innovate the field. The Youth PPI Café was designed to be both a part of an individual's recovery and an agent of change within NHS research culture—putting young people at the heart of every decision being made.

### 1.3 Aims

1. To explore how a diverse range of CYP could become interested and engaged in the research process.
2. To develop a model of meaningful engagement of CYP in research.
3. To codevelop a programme of meaningful research ‘with’ CYP, rather than ‘for’ them
  a. To work with young people to establish key priorities for research.
  b. To generate new youth‐focused research and opportunities, to further innovate the field of CYP mental health.

## 2 METHODS

### 2.1 Aim 1: To explore how a diverse range of CYP could become interested and engaged in the research process

To achieve our first aim, we began by carrying out two preliminary discussion forums with young people, to better understand some of the challenges that prevent meaningful and effective involvement. Between January and February 2020, we networked across SPFT to create a panel of approximately 20 young people (aged 21–25), the majority of whom were completing undergraduate internships within the trust, and some of whom had used services in the past. In recruiting to this panel, we undertook a pragmatic approach due to restrictive timescales, limited resources and residual challenges presented by the pandemic. In recognition of the perhaps limited representation of young people on this panel, we kept the group open to change going forward and adapted it to the needs of those eventually involved in the Youth PPI Café. This panel led on the priorities for setting up the Youth PPI Café, by exploring four key questions:

1. How do we engage young people?
2. Should we differentiate between age groups (i.e., child, adolescent, young adult) when looking at involvement with young people?
3. What are some of the best ways to communicate with young people?
4. What are some of the benefits and incentives we can offer for getting involved?

Researchers performed a process of thematic analysis to derive themes that may be relevant to young people looking to take part in research. An inductive approach was adopted when carrying out our analysis of these discussions.

### 2.2 Aim 2: To develop a model of meaningful engagement of CYP in research

The comments shared during discussions with our advisory group were used to inform the development of the Youth PPI Café. We began recruiting young people to this space between April and May 2020, from schools, charities and mental health services across Sussex. Though we had not anticipated launching this group during a global pandemic, we felt that it was important to continue with this group, especially given the isolating nature of the lockdown and the impact that we felt this was likely to have on CYP. Due to UK COVID‐19 restrictions on in‐person meetings in place at the time, we decided to hold sessions virtually via Zoom.

We kept much of the group open to change, so that the space could be codeveloped with those CYP involved. In doing so, we hoped to work with young people to achieve our second aim and develop a model of meaningful engagement of CYP in research.

### 2.3 Aim 3: To codevelop a programme of meaningful research ‘with’ CYP, rather than ‘for’ them

**Figure 1:**
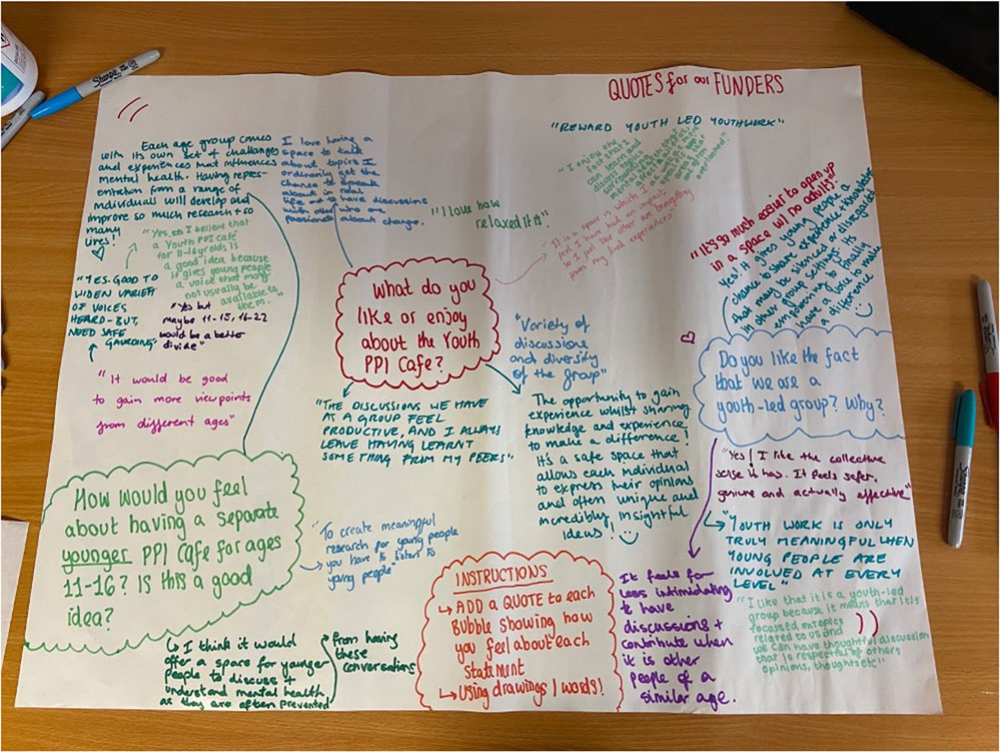
Image of a brainstorm from one of the quarterly feedback sessions, where young people shared their thoughts about being a part of the group for potential funders.

Though our third aim cuts across all of our work, the primary output comes through continuous collaboration with young people involved in the space. We aimed to meet at least every quarter for specific feedback sessions and discuss developments as a group so that the group grows in a way that our young members see fit (Figure 1).

We have worked with the young people involved to establish key priorities for research. The group decides what studies we explore and which areas of research we feel should be a focus, which directs our work. We hope that through these discussions, we can develop new youth‐focused research and opportunities in collaboration with young people, clinicians, commissioners and academics.

## 3 RESULTS

### 3.1 Aim 1: To explore how a diverse range of CYP could become interested and engaged in the research process

In our preliminary discussions with an advisory group, we set out to explore some of the challenges for meaningful and effective involvement with CYP. Several comments were shared during these discussions, from which three key themes were derived:

1. The *Social* experience of being a young service user and how this may influence engagement and involvement in research;
2. The *Emotional* experience of being a young person involved in research; and
3. Finally, any *Cognitive* barriers or differences in young people that we should consider when looking to involve young people in research.

For pragmatic reasons, these themes were derived by one researcher only (A.T.). The results of our analysis were later shared with the advisory group, to confirm that these themes upheld the messages they were trying to convey during our discussions.

#### 3.1.1 Social

A young person's social connections (e.g., friends, family, clubs and social categories) shape the way they view themselves and their interactions with others.^12^ In line with this, key themes around the social experience of being a young service user emerged, illuminating areas of interest that could be used to understand how we can better engage young people in research.

The advisory group discussed the importance of making youth involvement in research a collaborative effort, for example, engaging schools where young people spend most of their time, as well as services already working well with young people, to provide advice and resources regarding youth involvement. They also discussed some of the social aspects of working with young people. They felt that the facilitators of the group should be peers rather than figures of authority (e.g., a teacher, a clinician), to create the best social environment for young people to feel safe and be open.

*I like that it is a youth‐led group because it means that it is focused on topics related to us and we can have thoughtful discussions that are respectful of others' opinions, thoughts etc*. Youth PPI Café Member

The advisory group also wanted to emphasize that when working with young people, this should be a collaborative and respectful environment, with achievable, nontokenistic goals. One of the ways in which this could be achieved is by asking young people what they want to get out of these sessions and what they want to learn. They discussed how many young people are aware of and do discuss the world they are growing up in and the impacts this can have on well‐being, for example, the anxieties that come with climate change, and now more recently, global pandemics and lockdowns. It is vital that we hold space for this and consider broaching these topics when involving young people in research, perhaps during an informal catch‐up as a session begins. We cannot connect with young people in isolation—we must be prepared to listen and understand the worlds in which they live.

*I love having a space to talk about topics I ordinarily get the chance to speak about in real life and to have discussions with others who are passionate about change*. Youth PPI Café Member

#### 3.1.2 Emotional

When working with service users, especially those who are younger, the advisory group also felt that it was important to be aware of the emotional experience that is connected to involvement in research.^13^ Though experts by experience have a lot to offer, offering knowledge based on that experience can be potentially triggering. The advisory group explored some of the emotional experiences of being a young person today and discussed potential challenges or barriers to involvement.

They discussed ways to ensure that young people were aware that their involvement is purely for research and learning and that we are not offering psychological support. However, in recognition of the experience that can come with being involved in mental health research, we also wanted to make sure that we were adequately signposting service users to get support if needed. For example, we discussed having a mindfulness exercise, or even just a quick chat at the start and end of the group sessions, to debrief and destress from a distance. This would ensure that we are not disconnecting from the group immediately and allows sessions to end on a light note. The group also discussed how important it is to find ways to ensure that the young people we connect with virtually are adequately safeguarded. Though technology and social media are key in terms of engaging young people effectively, they provide new challenges when managing what could be a potentially triggering experience for some.

*I love how relaxed this space is*. Youth PPI Café Member

#### 3.1.3 Cognitive

Some interesting themes were also discussed regarding the cognitive capacities and capabilities of CYP and how we may need to alter our strategies of engagement when working with them in research and services. How a young person understands the world, and their place in it, is unique. Not only this, but the difference in cognitive capabilities between different people and also between different age groups (e.g., age 5–7 to 9–11) also varies dramatically,^14^ which can make it challenging to create an engaging and practical space for youth participation.

*The discussions we have as a group feel productive and I always leave having learnt something from my peers*. Youth PPI Café Member

The advisory group discussed the cognitive differences between different age groups of young people and the impact this may have on engagement. For example, the materials used within the group may need to differ according to age. We will also have to ensure that the language we use in resources, or that is present in videos, is accessible to all young people. They also discussed promoting this project amongst young people. One suggestion from the advisory group was to focus primarily on the visual element of our communications, so we can bridge the gap between different age groups—for example, using mascots that appeal to younger children and are not patronizing older children. There should be clear titles and headings to allow people to find the information they are looking for, quickly and easily. We should use targeted words such as ‘Inviting you’ and ‘young people’ or simply ‘we need you!’. The advisory group also discussed the different incentives we could offer to young people, in light of the academically structured world that they live in. For example, providing young people involved with a role title, and reference that can be put on a CV—‘Research Advisor’, as well as creating a LinkedIn page for the group so young people could list the project under their work experience. This has been highly successful in practice, and many of our young members gained their first paid employment following membership within the Youth PPI Café. The advisory group also discussed the different skills that can be gained during this project which may interest young people (e.g., communication, research, collaboration, leadership).

*The opportunity to gain experience whilst sharing knowledge and experience to make a difference! It's a safe space that allows each individual to express their opinions and often unique and incredibly insightful ideas!*. Youth PPI Café Member

### 3.2 Aim 2: To develop a model of meaningful engagement of CYP in research

Taking forward comments from the earlier sessions with our advisory group, the Youth PPI Café has been effective at engaging young people in research, through the codevelopment of a peer‐led space for involvement. This space has been hugely meaningful, both for the researchers and young people involved. Involving young people in research is known to aid recovery and can have an extremely positive impact on the well‐being of those involved.^2^ As well as developing skills in research, and participation, it has also developed confidence, and encouraged connections among young people with similar interests, passions, and experiences.^15^

The Youth PPI Café currently recruits young people aged 16–24 years, to work alongside dedicated clinicians and researchers, taking local research and service development projects forward. The young people come from Children and Young People's Services and Child and Adolescent Mental Health Services (CAMHS), local schools, colleges, and charities working with young people. Since our launch in May 2020, we have recruited over 100 young people within the Sussex area to the network, 20–30 of whom regularly attend sessions and engage in research. The majority of these young people were from White, middle‐class backgrounds. In recognition of this fact, increasing the diversity of this space has been a priority for the group. Those young members who identified as being from a minority background formed a working group to develop strategies to improve the accessibility of the Youth PPI Café for a broader range of young people. This study is ongoing.

#### 3.2.1 Sessions

Sessions were held every 4 weeks, from 5:30 to 7:00 pm, for a maximum of 15 young people per session. With more than 15 young people in a session, we found it became difficult to coordinate discussions and ensure that everyone could contribute equally, so a limit was set on the number of attendees. Each session explored a different research study or development project, with young people signing up for sessions online and joining via Zoom. Young people were given the option of having their camera on, or off, depending on what they were most comfortable with, though we did encourage cameras to be on where possible, for monitoring and safeguarding purposes.

We have held over 20 sessions in the last 18 months and participated in over 20 pieces of research and service development. Many studies were awarded funding as a result of their engagement with the Youth PPI Café and were supported by the young people involved.

*I'm unbelievably grateful to you all for all your work on this. I've written so many drafts and sent them to so many different researchers and clinicians, and by far this is the best feedback I've had on it so thanks. If I get the funding for this project, I'll be in touch with you all again, to let you know, (and hopefully recruit some of you as researchers/lived‐experience advisors to the project!)*. Researcher

Researchers who joined the sessions were asked to provide a short 1–2 sentence summary of their project beforehand, which was shared with the Youth PPI Café members at least a week in advance of the session. It was agreed this would be helpful, so members can adequately prepare themselves should a certain topic be particularly triggering. Attendance of sessions was not compulsory, and members were not treated any differently if they could not attend a particular session.

Following a session, minutes were typed up, and payment forms were collected so the young people in attendance can be paid for their time. The young people are sent the minutes, once complete, and asked to make any amendments before a final copy is passed on to the researchers who attended the session. Researchers are sent the final minutes and asked for their feedback. We look to stay in touch with researchers over time as the study progresses, to ensure that young people in the Youth PPI Café will be more than just tokenistic in their involvement in particular studies.

#### 3.2.2 Facilitators

In line with our initial discussions, facilitators of sessions had to be relatable and of a similar age to members. As such, one lead facilitator under the age of 24 (A.T.) hosted the sessions, supported by research assistants who took minutes and monitored the chatbox on Zoom. All activity both within, and outside of sessions, was coordinated by the lead facilitator for the Youth PPI Café. The lead facilitator received regular supervision from the PPI Team at SPFT, as well as all the necessary support and training in safeguarding and group facilitation. The lead facilitator and the research assistants met regularly to check in with each other ahead of sessions, to ensure that everyone felt supported in their role.

#### 3.2.3 The research

We initially provided young people with a range of studies which we as facilitators felt were appropriate but have since learned that there are certain areas which our young people feel must be prioritized, thus showing how over time, staff have listened to young people's voices and included them in decision‐making about the topics and areas that we focus on as a group. Priority areas include research on prevention and early intervention, as well as projects around service development within CAMHS. We have since focused our work on studies aligned with our core interests, in the hope that we can direct the field and establish key priorities for research.

*The young people were incredibly thoughtful in relation to my project, and I can tell that they have a lot of empathy and compassion for others who may be involved in research. Specifically, consideration of triggering imagery and making sure my information is dyslexia proof indicated this to me*. Researcher

#### 3.2.4 Adaptations

Since the launch, adaptations to sessions have been made to meet the needs of those group members involved, for example, each session now typically begins with an icebreaker, or casual check‐in, where the young people within the group can share a little bit about who they are and get to know each other better. This allowed us to generate added value through the social and emotional benefits associated with icebreakers, games and check‐in activities, providing a chance for young people to develop their social skills, and confidence and learn about one another. In each session, we also spend some time reflecting on the research explored in the previous session, as well as going over some feedback from the researchers who were involved. This gives us a chance to think about how our contributions will be taken forward. The process helped to create a sense of longevity and continuity for the young people involved, who could see and understand how their comments have been used by researchers, helping them to continue to feel part of an ongoing discussion about mental health and emotional wellbeing, as opposed to one‐off sessions with little follow‐up.

### 3.3 Aim 3: To codevelop a programme of meaningful research ‘with’ CYP, rather than ‘for’ them

At the core of the Youth PPI Café is a recognition that this space should be developed and led by young people. From assessment and formulation, continuing to evaluation we have included specific feedback sessions in our programme of work, meeting every quarter to discuss developments as a group, so that the group grows in a way which our young members see fit.

One significant example of this is in the development of our ‘Researcher Agreement’. Our researcher agreement is now used for every project carried out by the Youth PPI Café. This was written by the group as a whole, and outlines what the young people within the group expect from the researchers attending sessions, and what the researchers can expect from us. This has improved the relationship we have with the researchers attending and makes involvement more meaningful, and effective.

A further comment from our feedback sessions was the need to codesign a space for CYP aged 11–15 years to attend and engage in research. This will require co‐adapting the current 16–24 Youth PPI Café model, with this population, to suit their needs and appropriately engage them in research. By expanding the reach of the Youth PPI Café to this population of CYP, we will be able to support a wider range of relevant clinical research, including that focused on CAMHs which aims to prevent, promote, and treat mental ill‐health in the 11–24 population of young people. This 11–15 group is currently in development, to support the current transformation of CAMHS across the Southeast of England.

It is crucial to us as a group, that the Youth PPI Café offers more than just the opportunity to inform research, but also develops skills, experience and career development. We have supported current members of the Youth PPI Café in acting as ‘peer mentors’ for new, younger research advisors who wish to join. Equally, peer mentors can work with those who may be anxious about joining the space, or who may need extra support. This includes leading induction sessions, offering a Q&A before a session, and carrying out regular check‐ins with new members.

Another example of significant collaboration with young people includes the employment of one Youth PPI Café member who worked on the team to provide consultation and developmental support. E. P. joined the Youth PPI Café as a member at the launch in May 2020, before joining the team not long after. E. P. had this to say about his experience:

*As a member, I've watched the café grow and grow. While we've diversified our research topics and developed the provision that we offer, a key consistent factor throughout has been the participative and collaborative ‘safe space’ network that we've created. Our café has acted as a haven for young people to transform their lived experience into solution‐focused suggestions and measurable outcomes for research teams. Our research advisors have thrived in meetings due to the emphasis we put on youth leadership, which has allowed for a non‐judgemental and understanding dynamic between staff and the research advisors*.

*After joining the café in May 2020, I was invited to join the staff team in May 2021, supporting Abi with project developments and new activities. I've facilitated inductions and events for the café, which has helped me to gain experience in youth participation and has provided me with confidence when it comes to public speaking*.

*The café has greatly impacted on myself and my fellow youth research advisors, as it's provided a place for us to develop both socially and professionally. The café enables professional development from a young age, with youth advisors creating connections within both SPFT and the world of psychological research, which is excellent experience and has helped to kickstart both my career in youth work and my peer's careers in psychology and social research. The emphasis that the café places on CPD will serve our young people in good stead as they continue to develop their skills in participating in, advising on, or otherwise supporting mental health research*. Edward Peasgood

We are hoping to offer more paid staff roles to some of our young people who have been with us for over a year now. Roles will include helping the host to facilitate sessions with our new 11–15 Youth Café and carrying out outreach and recruitment work. We have also received interest in the role of a dedicated Social Media Assistant, who will manage our social media platforms, and write our newsletter. Another role may include coproducing, and designing a website, to create a collaborative online space connecting researchers, clinicians, and young people across the country. We hope to act as a national beacon for youth involvement in research, and this platform will allow the voices of young people to be heard on a wider scale. A website would also provide a great opportunity for us to offer development and leadership opportunities to young people, from outside the Café too. We hope to offer mentorship and signpost career pathways for all those involved. This study could also support and develop stakeholder interest, collaboration, and networking, including wider dissemination.

Our secondary aims (to work with young people to establish key priorities for research; to generate new youth‐focused research and funding opportunities, and to further innovate the field of CYP mental health) require further work, and if achieved, could place the Youth PPI Café at the centre of crucial advances in contemporary research for CYP. The Youth PPI Café has links across the Southeast and beyond, providing access to a range of cross‐disciplinary organizations carrying out research and providing data on a burgeoning scale. By leading a youth‐led model of engagement in research, one could further advance the field and fund meaningful and effective research.

## 4 DISCUSSION

The impact of the Youth PPI Café within the SPFT community has far surpassed expectations. The commitment, openness, and integrity of those young people involved in this space have shaped and developed the group into what it is today. The very nature of this space leaves it open to development, and much has been learned, shifted, and developed since its launch in May 2020. On the advice of those young people involved, we have been able to provide new opportunities for training, development, and involvement in the team, to lead the work being carried out within the remit of the Youth PPI Café.

Through our work over the next year, we hope to build partnerships and connections with other NHS trusts and forums that are passionate about improving the involvement of young people within our mental health services, and research. Building a network with key research funding bodies and organizations to ensure that Youth PPI is carried out appropriately, is a key priority for the group going forward. We hope to involve young people in the wider work of research and development, including inviting them onto sponsorship committees, involving them in training for professionals, and including their voices on funding boards and organizations to direct research for CYP.

### 4.1 Learning as we go

In getting to the stage we are at today, several challenges have been faced by both the Youth PPI Café facilitators and the members themselves. It is not without careful consideration of the group's experience and needs that we would have been able to overcome these challenges collaboratively.

Currently, there is no requirement for researchers to seek ethical approval for PPI, particularly in the design stage of research, but several ethical challenges still exist.^16^ For example, there are concerns that PPI is frequently undertaken in a tokenistic manner, an issue that has become embedded in the fundamental ethical principle of respect.^17^ In the Youth PPI Café, we try to encourage meaningful involvement and avoid working with those for whom involvement with young people is more of a ‘tick‐box’ activity. We ask researchers to provide feedback on their experience within the group and keep us updated as to where our comments are used, and their impact, as well as developments throughout the project itself. It became evident in the first few months of working with different researchers, that it was not always the case that we would hear back from them after they participated in the group. Be this the result of an over‐capacitated NHS system, or the researchers themselves, we were adamant to make a change. Our ‘Researcher's Agreement’ which is now given to researchers before they attend the sessions, outlines the responsibilities of the group, and the researchers themselves, in working together. It has been hugely successful in lieu of research ethics, in ensuring mutual respect between Youth PPI Members and researchers in attendance.

Another core challenge of this study has been including those young people who are unable to access a virtual session easily, either due to a lack of digital skills or access to the technology required. Limited by COVID restrictions and resourcing, we have remained within the virtual sphere of Zoom when carrying out our sessions. However, we are aware that in doing so, and in moving our communications online to a website, we are excluding a large population of young people who desire to be involved in research, but who might not have the tools or resources to do so. We hope to reach out to this demographic of young people over the next year and ask them what they would prefer in a space like the Youth PPI Café. How can we adapt it to meet their needs and ensure that their voices are heard amongst those young people who are better able to participate? We are seeking to launch a new 11–15 Youth PPI Café in the coming months, which will be held in person and should facilitate inclusivity of CYP from minority groups.

We also faced several financial and resourcing challenges throughout the development and running of the Youth PPI Café. Despite its positive impacts, there was a substantial lack of funding available to support meaningful PPI activities, processes, and expansion, limiting the scope of the project as a whole. This is a familiar challenge for those working in PPI within the NHS. Indeed, many agree that PPI has a low relative priority for funders, which significantly hinders the beneficial impacts of user involvement on research.^18^ At a certain point in the project, the demand from young people looking to be a part of the Youth PPI Café became much greater than our capacity. As such, we couldn't offer the space to everyone, as we had originally hoped. We offered alternative methods of involvement (such as online anonymous surveys) but these were ultimately voluntary, and it was disappointing for many young people when they were unable to attend a session and share their views. A lack of funding also hindered the expansion of paid positions in our team, which were developed for young people looking to support the work of the Youth PPI Café as it expanded. Though one young member took up a voluntary role within the team, we are still seeking funding for further paid positions for our young people. Of course, reduced funding and capacity will always present a challenge to those working within the NHS, but it is clear that much more practical support is needed for PPI, including funding, time, and the opportunity to share practice across organizations working with young people in a research context.^19^ PPI members can offer unique and valuable insights which have the potential to make research more effective, credible, and cost‐effective, and funding opportunities should reflect this.^19^, ^20^

### 4.2 Overcoming challenges

Several of the challenges faced within the Youth PPI Café were overcome collaboratively, with the young members, and peer mentors from the group. Our researcher agreement is one example—an idea developed by the young people involved, to combat the lack of respect and communication from a minority of researchers attending our space. In many current debates about PPI in research, the dominant discourse is one in which researchers hold the power and contributors do not,^19^ and this can be even more apparent in the case of PPI with young people. By creating a space led by young people, for people, we were able to give power back to our young research advisors and approach any challenges together. E. P., who offered consultation and developmental support for the Youth PPI Café was integral to moving the space forwards and finding solutions to challenges we faced in recruiting new members, seeking funding, and building a space for younger people (aged 11–15) to also share their views, and become involved in research. Though ultimately, we relied on adults within SPFT to provide funding, by including young people within our team and offering them a chance to be involved in discussions about the running of the group and the research we discuss, we were able to create a PPI group where young people could confidently use their lived experience to shape mental health research.

This experience has also demonstrated the need to develop a quality assessment tool to guide researchers on how to ethically conduct PPI with young people specifically. Young people cannot be solely relied upon to change involvement practices themselves. Instead, researchers must also reflect on their own practice, and ensure they are working to create an environment where young people can make meaningful contributions to mental health research. Researchers have embedded the practice of PPI inconsistently, a fact that is at the heart of many of the challenges we experienced as a group. By developing more meaningful frameworks of involvement with young people, one could maximize the real value PPI brings to research.^21^

## 5 CONCLUSION

There is still much to be done to improve youth involvement in mental health research. Throughout this project, and across our core aims, Youth PPI has benefited not just the research, but also the young people involved. Involving youth meaningfully in research has the potential to inform studies at a macro‐ and microlevel, enabling positive change within research and within the systems that support CYP.

## CONFLICT OF INTEREST

The authors declare no conflict of interest.

## Data Availability

Data availability is not applicable to this article as no new data were created or analysed in this study.
